# Impacts of organic materials amendment on the soil antibiotic resistome in subtropical paddy fields

**DOI:** 10.3389/fmicb.2022.1075234

**Published:** 2023-01-24

**Authors:** Zongming Li, Jupei Shen, Fangfang Wang, Meihui Wang, Jianlin Shen, Yong Li, Qihong Zhu, Jinshui Wu

**Affiliations:** ^1^Key Laboratory of Agro-Ecological Processes in Subtropical Region and Changsha Research Station for Agricultural and Environmental Monitoring, Institute of Subtropical Agriculture, Chinese Academy of Sciences, Changsha, China; ^2^College of Resources and Environment, University of the Chinese Academy of Sciences, Beijing, China; ^3^School of Geographical Sciences, Fujian Normal University, Fuzhou, China; ^4^State Key Laboratory of Crop Genetics and Germplasm Enhancement, College of Resources and Environmental Sciences, Nanjing Agricultural University, Nanjing, China

**Keywords:** organic materials, biochar, antibiotic resistance genes, bacterial community, paddy soil

## Abstract

The organic material amendment has been proven to change the soil antibiotic resistance genes (ARGs) profile, which may threaten human health through the food chain, but the effects and mechanisms of different organic materials on ARGs in paddy soils are less explored. In this study, a field experiment was set up with the treatments of conventional chemical fertilization (NPK) and common organic material amendment [rice straw (RS), swine manure (SM), and biochar (BC)] to explore the effects and mechanisms. In total, 84 unique ARGs were found across the soil samples with different organic material amendments, and they conferred resistance to the major antibiotic classes. Compared with NPK, SM significantly increased the detected number and relative abundance of ARGs. A higher detected number of ARGs than NPK was observed in BC, whereas BC had a lower relative abundance of ARGs than NPK. Compared with NPK, a detected number decrease was observed in RS, although abundance showed no significant differences. Compared with other treatments, a higher detected number and relative abundance of mobile genetic elements (MGEs) were observed in BC, indicating a higher potential for horizontal gene transfer. There were significantly positive relationships between the relative abundances of total ARGs and MGEs and the bacterial abundance. The network analysis suggested the important role of MGEs and bacterial communities in shaping the ARGs profile. Mantel test and redundancy analysis (RDA) suggested that soil carbon, nitrogen, and C/N were the major chemical drivers of the ARGs profile. The risk of ARGs spreading to the food chain should be considered when applying SM and biochar, which shifted the ARGs and MGEs profiles, respectively. Pre-treatment measures need to be studied to reduce the dissemination of ARGs in paddy fields.

## 1. Introduction

The emergence and prevalence of antimicrobial resistance (ARGs) pose a major threat to public health (WHO, 2014); it has gained numerous concerns. The environmental microbes that carried ARGs had similar gene sequences to clinical pathogens, suggesting the potential transmission of ARGs from the environment to the pathogens of human beings or *vice versa*. Most importantly, more and more evidence proved that ARGs in manured soil can be transferred to the phyllosphere of vegetables (Zhang et al., 2019). Thus, the bloom and dissemination of ARGs in agroecosystems have potential risks to agricultural production and food security (He et al., 2020). Furthermore, native soil microbes could acquire ARGs *via* horizontal gene transfer mediated by mobile genetic elements (MGEs) from exogenous microorganisms (Chen et al., 2017). Thus, it is important to explore the antibiotic resistome in the agricultural ecosystem for assessing the potential risk.

Antibiotic resistance is an ancient and natural phenomenon (D’Costa et al., 2011; Shen et al., 2019), and human activities such as livestock production, composting, and manure fertilization have put selective pressure on antimicrobial resistance in various environments (Zhu et al., 2013; Forsberg et al., 2014). Soil is probably the largest habitat for microbes and one of the largest reservoirs of ARGs (Forsberg et al., 2012; Nesme et al., 2014), especially agricultural soil, which suffered from human activities. ARGs were widely detected in paddy fields (Zhao et al., 2020), vegetable farmlands (Xu et al., 2021), and even in the phyllosphere of vegetables (Chen et al., 2018; Zhou et al., 2019). In recent years, many studies have documented that livestock manure, reclaimed water, sewage sludge, and heavy metals play pivotal roles in profiling the patterns of ARGs in the soil environment (He et al., 2020). Agricultural soils play a critical role in sustaining crops and the food supply. To promote food production and sustainable development, chemical fertilizers and organic materials (for instance, livestock manure, straw, and biochar) were widely applied in croplands (Tiedje et al., 2019). Livestock manure application could introduce the ARGs that they carried into the soil and place selective pressure on soil indigenous stocktickerARG-bearing microbes *via* residual antibiotics or (and) heavy metals (Chen et al., 2017, 2019; Han et al., 2018). Fertilization not only influences soil physicochemical properties such as pH, available nitrogen, and soil organic matter contents but also affects microbial diversity, abundance, and community structure (Xie W. et al., 2018; Sun et al., 2019; Chen P. et al., 2021). A great number of studies have been conducted to assess the impacts of fertilizer application on the soil microbiome and function guilds (Dai et al., 2018; Jia et al., 2020). Organic materials, such as crop straw, straw-derived biochar, and swine manure (SM), are usually applied with chemical fertilizers to croplands for soil fertility improvement. Nevertheless, the impacts of different organic materials input on the antibiotic resistome in paddy fields are still less explored.

Different fertilizers make distinct contributions to the structure and function of microbial communities in agricultural soils. Long-term overuse of chemical fertilizers decreased soil pH and then shifted the structure of the soil bacterial community (Dai et al., 2018), even inhibiting the activity of bacteria (Lin et al., 2016; Jia et al., 2020). Additionally, the influences of chemical fertilizers on the relative abundance of ARGs in different tillage systems were inconsistent in previous reports (Wang F. et al., 2018; Han et al., 2021). Organic fertilizers, such as livestock manure, composting, and sludge, were widely amended into paddy soil. Consequently, the microbes carrying ARGs from animals or humans were introduced into the soil and shifted the structure of native microbial communities in the soil (Macedo et al., 2021). Generally, manure application promotes the propagation of ARGs, for instance, genes conferring resistance to sulfonamide (Tang et al., 2015; Lin et al., 2016; Xu et al., 2021). Recently, biochar was applied to improve soil fertility and reduce soil pollutants, including antibiotics and heavy metals (Ye et al., 2016; Wang S. et al., 2021). Biochar derived from various organic materials such as rice straw (RS), wheat straw, and maize straw can increase soil fertility and supply a unique habitat for microbes, then directly or indirectly change the abundance and diversity of microbes (Zhang G. et al., 2021). Previous studies showed that biochar addition reduced the relative abundance of ARGs in arable soils (Ye et al., 2016; Duan et al., 2017), while some found that biochar containing heavy metals could increase the relative abundance of ARGs (Ding et al., 2019). RS incorporation is another common agricultural practice, which significantly influences bacterial community composition and abundance (Zhang S. et al., 2021). To some extent, soil bacterial abundance and communities shifting accounts for the ARGs profiles feature. Organic materials input influences the microbial composition and abundance, which in turn impacts the shape of ARGs. It is essential to evaluate the effects of different organic materials input on the ARGs profile in the soils for further risk assessment. In this study, a field experiment was conducted with the aims (1) to determine the effect of different organic materials amendment on antibiotic resistome and bacterial communities in paddy soils and (2) to explore the underlying mechanisms of the effects of organic materials on the paddy soil resistome.

## 2. Materials and methods

### 2.1. Sampling and DNA extraction

The field experiment was conducted at the field experiment station (113° 19′ 52″ E, 28° 33′ 04″ N) of the Institute of Subtropical Agriculture, Chinese Academy of Sciences, which is located in Changsha County, Hunan Province in southern China. The study site had a subtropical humid monsoon climate, with an annual mean precipitation of 1,330 mm and an annual air temperature of 17.5^°^C (Shen et al., 2014).

The treatments of the field experiment with the double rice cropping included CK (without nitrogen fertilizer), NPK (NPK chemical fertilizers only), RS (chemical NPK fertilizers combined with RS at a rate of 6 t dry matter ha^–1^ in each rice season), SM (chemical NPK fertilizers combined with SM at a nitrogen supply ratio of 1:1), and BC (chemical NPK fertilizer combined with straw-derived biochar at a rate of 24 t dry matter ha^–1^ applied only once). Except for the CK treatment, all the treatments had the same N fertilizer application rate in each rice season. The experimental plots for each treatment were set up in triplicate. The chemical NPK fertilizers were composed of urea (120 kg and 150 kg N ha^–1^ in early and later rice seasons, respectively, with the application rate for basal, tillering, and panicle fertilizers at a ratio of 5:3:2), superphosphate (75 kg P_2_O_5_ ha^–1^ used as basal fertilizer), and potassium chloride (100 kg K_2_O ha^–1^ used as basal fertilizer). The field experiment started in 2012.

Topsoil samples (0–20 cm) for all the plots of the treatments were collected with a sterilized shovel after harvest in the late rice season of 2019. One part of the soil samples for each plot was put in liquid nitrogen immediately for transport to the lab and stored at –80^°^C until use, and another part of the soil samples was put on ice for transport to the lab and stored at 4^°^C until use. Chemical properties of soil samples, including total nitrogen (TN), total phosphorus (TP), total organic carbon (TOC), available phosphorus (AP), nitrate (NO_3_^–^-N), and ammonium (NH_4_^+^-N) were measured as described previously (Wang C. et al., 2021). The total microbial DNA of the soil was extracted using a DNeasy PowerSoil kit from 0.5 g fresh soil according to the manufacturer’s instructions (Qiagen, Inc.). The concentration of extracted DNA was detected using NanoDrop One, and DNA was stored at –80^°^C until use.

### 2.2. Quantitative PCR analysis

A high-throughput qPCR (HT-qPCR) was used to quantify the ARGs and MGEs in soil samples. The array included 384 primers targeting ARGs (319) and MGEs (57); additionally, the taxonomic marker genes were included in the array (Stedtfeld et al., 2018; Kanger et al., 2020; Pu et al., 2020). All reactions were performed in the Takara SmartChip real-time PCR system, as described previously. Three technical replicates were performed for each sample, and a non-template negative control was included in each HT-qPCR run (Kanger et al., 2020). A threshold cycle (Ct) of 31 was used as the detection limit, and only all three replicates with Ct lower than 31 were regarded as genes detected in that sample. The relative abundance of detected ARGs was calculated using a previously reported formula (Han et al., 2021).

Real-time quantitative PCR was performed to quantify total bacterial 16S rRNA gene copies using a primer set as previously described (Pu et al., 2020). The 25-μL reaction mixture contained 12.5 μL of premixture (Takara, Japan), 0.5 μL of each primer (10 μM), 1 μL of DNA (∼10 ng μL^–1^), and 10.5 μL of RNase-free water. The bacterial 16S rRNA gene primer set and amplification condition were the same as HT-qPCR (Pu et al., 2020).

### 2.3. Bacterial 16S rRNA gene amplicon sequencing and taxonomic analyses

The bacterial composition in different fertilization practices was surveyed by prokaryotic 16S rRNA gene amplicon sequencing with primer pair 338F and 806R, which target the V3–V4 variable region of the 16S rRNA gene (Xu et al., 2016). The amplicon sequencing was performed on the Illumina MiSeq System (PE300) by Shanghai Majorbio Bio-Pharm Technology Co., Ltd.

Microbiome bioinformatics was performed with qiime2 2019.7 (Bolyen et al., 2019). The raw sequencing reads were demultiplexed and quality filtered using the q2-demux plugin, followed by denoising with DADA2 (Callahan et al., 2016) (*via* q2-dada2). Taxonomy was assigned to ASVs using the q2-feature classifier (Bokulich et al., 2018) against the SILVA taxonomy database (release 132) based on a 97% sequence similarity threshold (McDonald et al., 2012). The raw sequences have been deposited in the NODE.^1^

### 2.4. Statistical analysis and data visualization

One-way analysis of variance (ANOVA) followed by the Student-Newman–Keuls test was carried out in SPSS 22.0 to compare the difference in diversity and the relative abundances of ARGs and MGEs across different treatments. *P* < 0.05 was considered to be statistically significant. The relative abundance of *aac(3)-Via* is one to three orders of magnitude higher than that of other detected genes, which will seriously affect the analysis results, so *aac(3)-Via* was discarded during analysis. A Venn diagram was generated to visualize the number of shared ARGs between different treatments using the Evenn (Chen T. et al., 2021). The difference in the relative abundance of ARGs and the community compositions of bacteria among different fertilization approaches was visualized by principal coordinates analysis (PCoA) based on the Bray–Curtis dissimilarity distances using the “vegan” package in R (Oksanen et al., 2020). A Mantel test was conducted to assess the correlations between soil properties, MGEs, bacterial abundance, bacterial diversity, and ARGs based on Bray–Curtis dissimilarity matrices with 999 permutations using the “linkET” package in R. Furthermore, transformation-based redundancy analysis (RDA) was carried out to explore the relationship between the composition of ARGs and soil chemical and biological parameters using the “vegan” package in R (Oksanen et al., 2020).

Networks were illustrated to explore the co-occurrence pattern between MGEs, bacterial taxa, and ARGs based on the Spearman correlation coefficients. The Spearman correlation coefficient (ρ) > 0.6 and *P* < 0.01 were regarded as statistically robust correlations (Li et al., 2015). The correlation coefficient matrices were imported into Gephi 0.92 for visualization (Bastian et al., 2009), and the network topology was explored by the Frucherman-Reingold algorithm.

## 3. Results

### 3.1. Diversity and abundance of ARGs under different organic materials input

**FIGURE 1:**
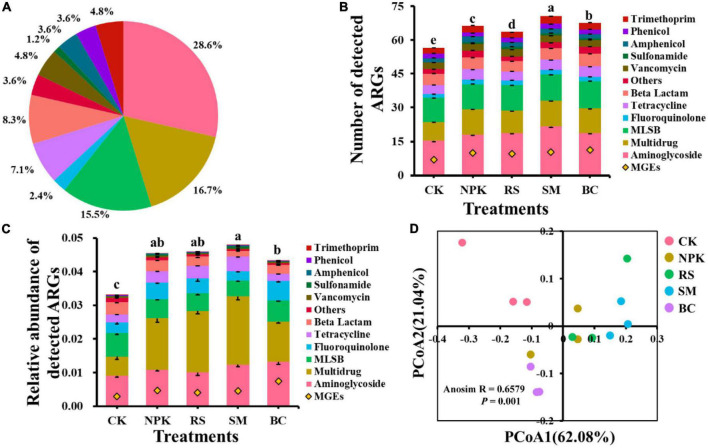
Classification of ARGs based on the antibiotic to which they confer resistance **(A)**. Detection number **(B)** and relative abundance **(C)** of ARGs categorized by antibiotic type detected in different management practices. The PCoA analysis is based on the relative abundance of ARGs using Bray–Curtis distances **(D)**. MLSB, macrolide-Lincosamide-Streptogramin B. Different letters above the bars indicate a significant difference (*P* < 0.05) across different treatments.

**FIGURE 2:**
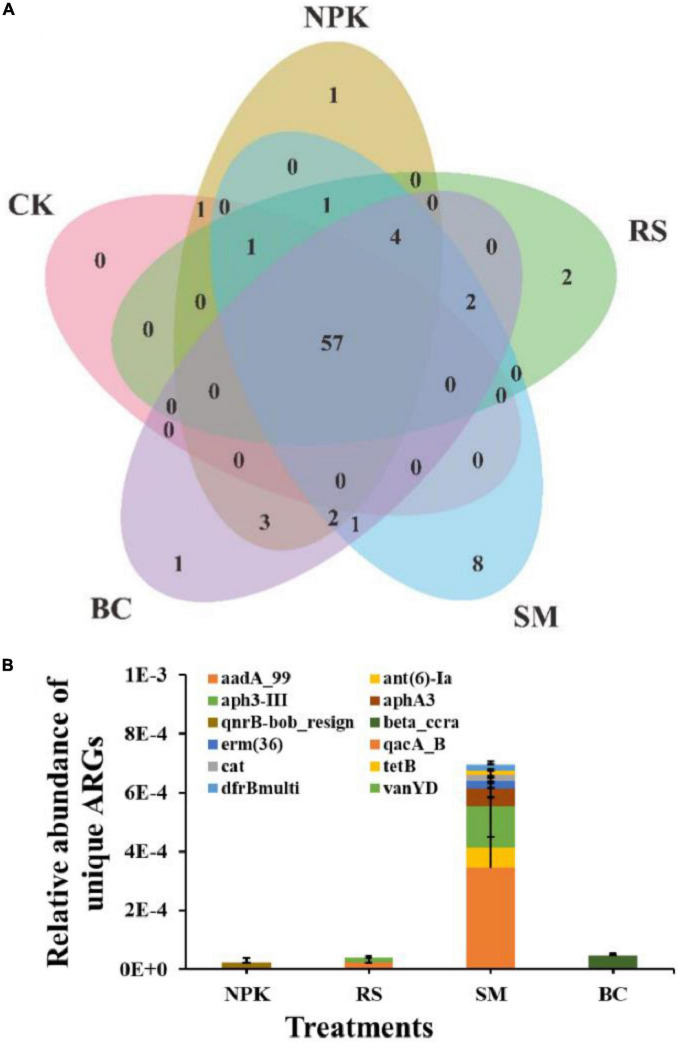
A Venn diagram showing the ARG numbers shared by different treatments **(A)**, and the relative abundance of unique genes detected in different treatments **(B)**.

**FIGURE 3:**
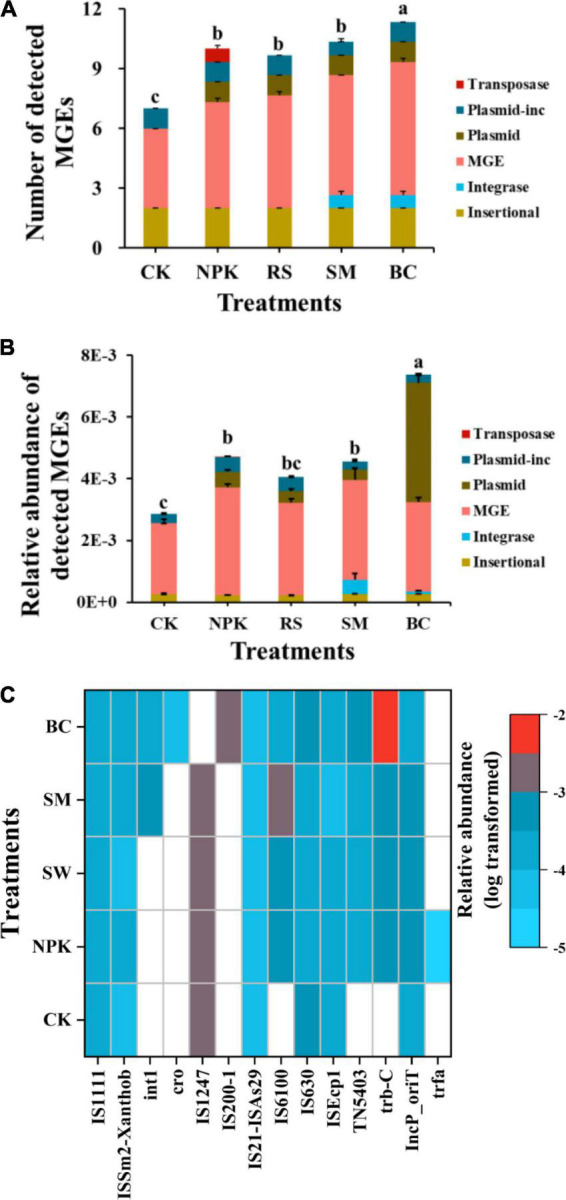
Detection number **(A)** and relative abundance **(B)** of MGEs categorized by MGE type detected in different management practices and the relative abundance of individual MGEs detected in soil samples **(C)**.

A total of 84 ARGs and 14 MGEs were detected across all samples (Figure 1A). These detected ARGs conferred resistance to the major eight antibiotics commonly used in the clinic or husbandry: aminoglycosides, beta-lactams, fluoroquinolones, MLSB (macrolides-lincosamides-streptogramins B), sulfonamides, tetracyclines, vancomycin, and others. Compared to CK, nitrogen and organic materials input significantly increased the number of detected ARGs in paddy soil regardless of the organic material types (Figure 1B). Under the treatments with the same nitrogen application rate, SM detected the highest number of ARGs (*P* < 0.05), followed by BC, NPK, and RS, in descending order. The three most frequently detected ARG classes, conferring resistance to aminoglycoside, multidrug, and MLSB, accounted for 28.6, 16.7, and 15.5% of the total number of detected ARGs, respectively. Interestingly, the plasmid-mediated colistin resistance determinant *mcr-1* gene was detected in all samples, which was first detected in animal guts and conferred resistance to the “last resort,” polymyxin. No genes conferring resistance to sulfonamide were detected in CK, and only one was detected in other treatments. In addition, the number of shared ARGs among different treatments was shown in the Venn diagram (Figure 2A). Nitrogen and organic material applications changed the ARGs profiles in soils. Compared to CK, the number of unique ARGs detected in different organic materials ranged from 1 to 8 (Figure 2). There were 57 ARGs found in all the treatments. In terms of MGEs, the detected number for the BC treatment was significantly higher than that for other organic materials treatments (*P* < 0.05), while no significant differences were observed in the detected number of MGEs among NPK, RS, and SM (Figure 3A). The HT-qPCR array detected a wide type of MGEs, including two insertional sequences, one integrase, two plasmids, and one transposase (Figure 3C).

For the relative abundance of ARGs, the genes conferring resistance to aminoglycoside, multidrug, and MLSB shared the highest relative abundance among all the treatments (Figure 1C), with the relative abundance in the range of 63.3–66.8, 7.78–7.5, and 4.08–9.52%), respectively, which, in total, accounted for 80.6–86.9% of the total abundance of ARGs. Compared to CK, the treatments with nitrogen fertilizer application significantly increased the total relative abundance of ARGs (*P* < 0.05). Under the same rate of nitrogen input, the total relative abundance of ARGs was the highest in the SM treatment and the lowest in the BC treatment (*P* < 0.05), and no significant differences were observed among RS, NPK, and BC (*P* > 0.05). There was no significant variance observed in the relative abundance of ARGs conferring resistance to amphenicol, trimethoprim, and vancomycin across all treatments, whereas variance was observed in other classes of ARGs among all treatments (Supplementary Table 2). For example, a higher total relative abundance of genes refer resistance to multidrug was observed in SM and RS than in other treatments under the same nitrogen input rate (*P* < 0.05), and BC was lower than NPK (*P* < 0.05). Regarding the relative abundance of total MGEs, an obvious increment was observed after nitrogen input and organic materials input (Figure 3B). Under the same nitrogen rate application, BC owned the highest detected number and relative abundance of total MGEs than other treatments (*P* < 0.05), while no significant variances were observed among other organic materials treatments. Divergent compositions of MGEs were observed among different treatments with different relative abundances (log-transformed, Figure 3C).

Differences in the comprehensive composition of ARGs among different treatments were assessed further by PCoA analysis based on the Bray–Curtis distance of the relative abundance of ARGs. The results showed that soil samples from CK were distinctly separated from others amended with organic materials (Figure 1D). Approximately 83.1% of the total variation was explained by the first and second axes of the ARGs structure.

### 3.2. Bacterial abundance, diversity, and community structure

The absolute abundance of the 16S rRNA gene in CK was 4.48 × 10^9^ copies per gram of dried soil, which was significantly lower than those of the treatments with nitrogen fertilizer application. There was no significant difference in the absolute abundances of the 16S rRNA gene among the treatments with the same rate of nitrogen input (Supplementary Figure 1). For the bacterial Shannon-Winner index, there was no significant difference across all the treatments (Supplementary Figure 1).

Across all soil samples, the dominant bacterial phyla were *Proteobacteria* (33.3%), *Chloroflexi* (17.0%), *Acidobacteria* (10.8%), and *Nitrospirae* (9.2%), accounting for more than 70% of the total bacterial sequences (Supplementary Figure 1). The ANOVA analysis was performed to find the variation in the relative abundance of bacterial phyla across all treatments. Only *Nitrospirae* and *Bacteroidetes* showed statistical differences across all five treatments, while the left phyla showed no significant differences across the treatments. Briefly, the relative abundance of *Nitrospirae* was the highest (*P* < 0.05) for the BC treatment as compared with other treatments with nitrogen fertilizer application, and there were no significant differences among the treatments of NPK, RS, and SM. Under the same nitrogen input rate, the relative abundances of *Bacteroidetes* for the treatments with organic materials input (RS, SM, and BC) were lower than that of NPK (*P* < 0.05). In addition, the PCoA based on the Bray–Curtis distance metrics showed no significant differences in bacterial community composition among all fertilization practices (Supplementary Figure 1).

### 3.3. Correlations between ARGs and MGEs

**FIGURE 4:**
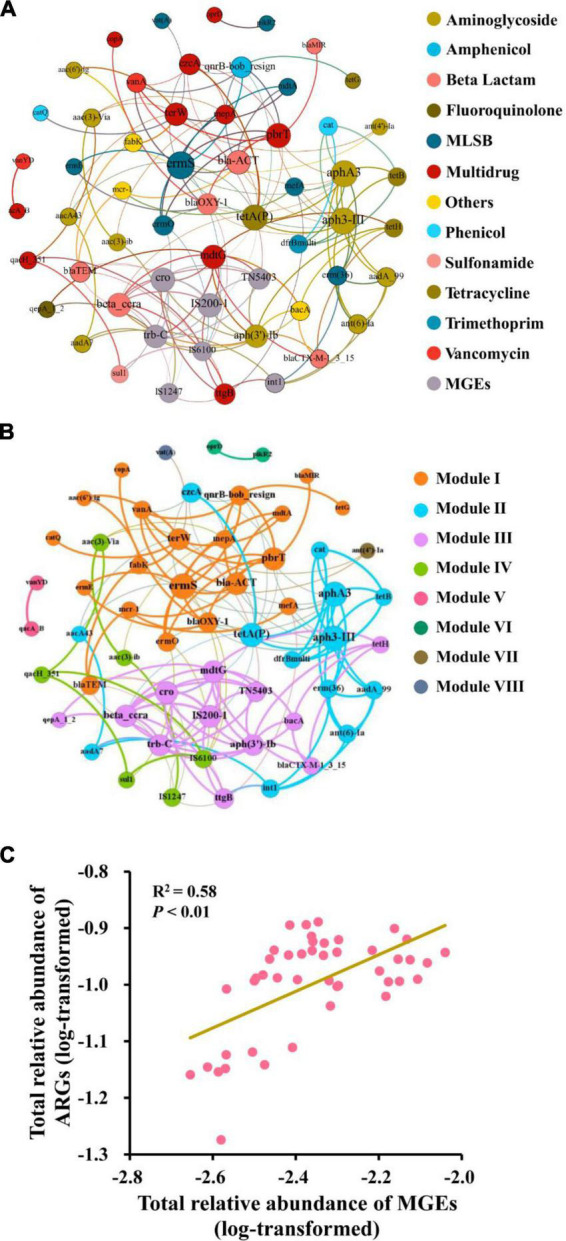
Correlation between ARGs and MGEs. Network illustrating the co-occurrence patterns among the detected ARGs and MGEs across all the soil samples. The nodes with different colors represent different categories of ARGs and MGEs **(A)** or different modules **(B)**. Correlation between the relative abundance of total ARGs and MGEs **(C)**.

The network was composed of 57 nodes (50 ARGs and 7 MGEs) and 151 edges (Figure 4A). A total of 19 ARGs co-occurred with MGEs, and some of them were highly detected frequencies and relative abundances, such as *ermS*, *tetA(P)*, and *mdtG*. The clusters of nodes (modules) were found in the network, and there were eight modules. The nodes that connected intensively with each other were regarded as the “hubs” and used as the indicators of co-occurring ARGs in the same module. In the largest three modules (I, II, and III), the gene *ermS*, *tetA(P)*, and *mdtG* were the hubs of these major modules, respectively (Figure 4B). Mostly, four MGEs (*IS200-1*, *TN5403*, *trbC*, and *cro*) are located in Module III. Furthermore, Pearson correlation analysis indicated that the relative abundance of total ARGs had a significantly positive relationship with the relative abundance of total MGEs (*r*^2^ = 0.58, *P* < 0.01) (Figure 4C).

### 3.4. Relationships between bacterial communities, soil properties, and ARGs

**FIGURE 5:**
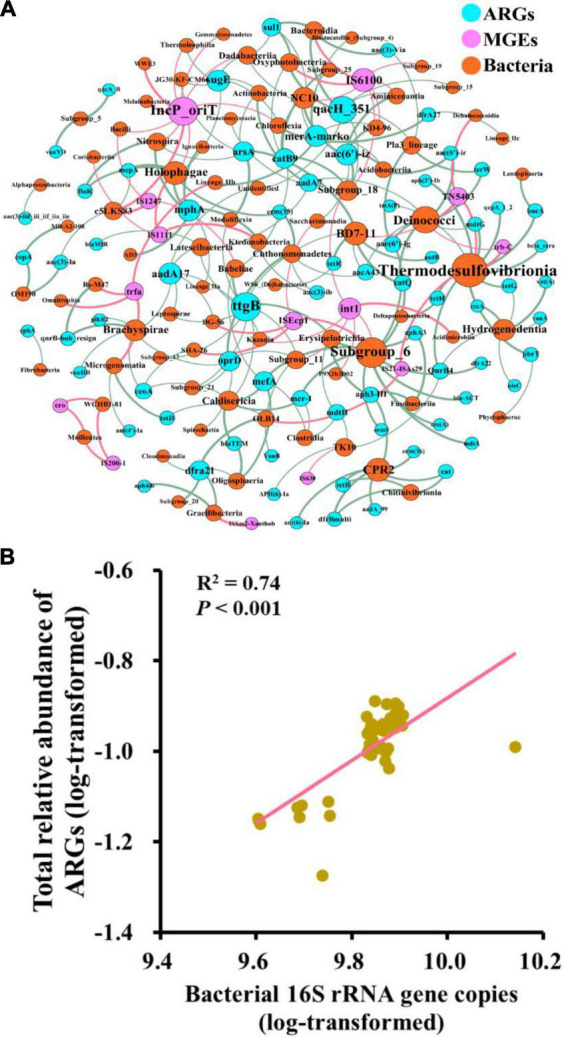
The network of co-occurrence patterns among ARGs, MGEs, and bacterial taxa at the class level **(A)** and the correlation between 16S rRNA gene copies and the relative abundance of total ARGs **(B)**.

**FIGURE 6:**
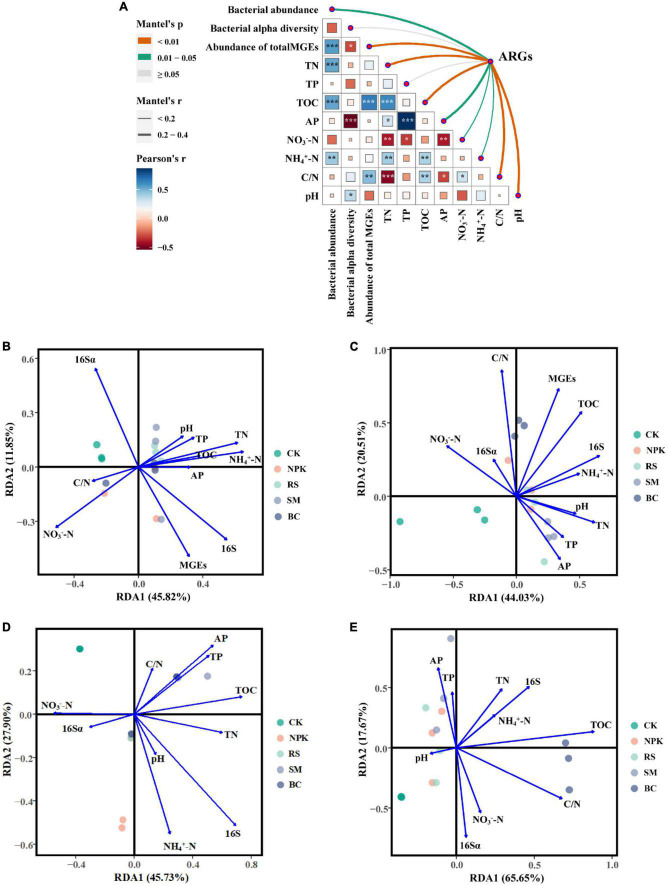
Pairwise comparisons of environmental factors (soil properties, bacteria, and MGEs) are shown, with a color gradient denoting Pearson’s correlation coefficient **(A)**. The composition of ARGs was related to each environmental factor by Mantel tests. Edge width corresponds to the Mantel’s r statistic for the corresponding distance correlations, and edge color denotes the statistical significance based on 999 permutations. Redundancy analysis (RDA) of the effects of environmental factors on detected number **(B)** and ARGs abundance **(C)** across all treatments, and the effects of environmental factors on MGEs detected number **(D)** and abundance **(E)** across all treatments. In all figures, C/N means the ratio of soil total carbon and total nitrogen, TOC means soil total carbon, TN means soil total nitrogen, TP means soil total phosphorus, AP means soil available phosphorus, NO_3_^–^-N means soil nitrate nitrogen content, and NH_4_^+^-N means soil ammonium nitrogen content. **(B–E)** The 16S means the bacterial abundance, and the 16Sα means the bacterial alpha diversity (Shannon-Winner index).

The co-occurrence patterns between ARGs and bacterial taxa (class level) were studied using the network analysis based on the Spearman correlation relationships (ρ > 0.6, *P* < 0.01). The non-random co-occurrence patterns between bacterial taxa and ARGs could provide indirect evidence of potential host information for ARGs. This network was composed of 168 nodes (73 ARGs, 14 MGEs, and 81 bacterial taxa) and 231 edges. As shown in Figure 5A, *Thermodesulfovibrionia* (*Nitrospirae*) connected with 10 ARGs and three MGEs, which was the largest bacterial node, followed by *Subgroup_6* (*Acidobacteria*) and *Deinococci* (*Deinococcus-Thermus*). *For ARGs, ttgB connected with 10 bacterial taxa, which was the largest node of ARGs, followed by qach_351. In terms of MGEs, IncP_oriT was the largest node of MGEs, followed by IS6100 and int1*. Furthermore, bacterial 16S rRNA gene copies showed a significantly positive correlation with the relative abundance of total ARGs (*r*^2^ = 0.74, *P* < 0.001) (Figure 5B), and the Mantel test showed that bacterial 16S rRNA gene copies had a significant positive correlation with the composition of ARGs (*r* = 0.25, *P* < 0.05) (Figure 6A). In addition, MGEs’ abundance, soil TN, TOC, C/N (C/N ratio), and pH also showed significant correlation with ARGs composition by mantel test (*P* < 0.01, Figure 6A). RDA analysis was performed to further verify and identify the main drivers of ARGs composition. For all treatments, the RDA analysis explained 87.1% of the total variability in the ARGs structure, and the first two axes account for 64.54% (Figure 6C). ARGs in the CK were separated from those in the nitrogen input treatments along the first axis. The contribution of bacterial abundance (16S rRNA gene copies) and abundance of total MGEs to the variation of ARGs accounted for 12.43 and 10.25%, respectively (*P* < 0.05). TN, TOC, and C/N were the top three soil properties that contributed to the abundance of ARGs, accounting for 11.38, 11.31, and 11.91%, respectively (*P* < 0.05). TN also made a great contribution to the detected number of ARGs (11.26%, Figure 6B). Furthermore, TOC was the main influencing factor of MGEs detected number (15.75%, Figure 6D) and abundance (29.37%, Figure 6E). The abundance of bacterial and MGEs were the main biotic factors shaping the ARG patterns, while TN, TOC, and C/N were the main abiotic drivers. The bacterial abundance played the most important biotic role in shaping the abundance of ARGs, while the MGEs’ abundance played the most important role in profiling the number of ARGs.

## 4. Discussion

### 4.1. Effects of nitrogen and organic materials on antibiotic resistome in paddy soil

In this study, nitrogen fertilizers induced dramatic shifts in soil properties and microbial communities and also played a crucial role in shaping soil antibiotic resistome. In the previous studies, the increase and/or decrease in ARGs abundance induced by chemical fertilizer use were observed, even with a limited effect. For example, the *tetA* gene abundance in grassland was enhanced by nitrogen input (Nõlvak et al., 2016), while depletion of most *tet* genes was observed in the paddy-upland rotation system (Lin et al., 2016) with chemical fertilizer application as compared with a control with no fertilizer applied. In this study, the diversity and abundance of ARGs were higher in NPK than in CK, and the compositions of ARGs were obviously different between the two treatments. That implied an important role for nitrogen application in shaping ARGs pattern. The unique gene detected in NPK (*qnrB-bob_resign*) and the gene shared by CK and NPK (*terW*) could be considered as chemical fertilizers input-induced. Compared to CK, although only a slight increment in soil TN was observed in NPK (Supplementary Table 1), this would lead to an increase in bacterial abundance, especially nitrogen-favored organisms, and then affect resistome composition (Forsberg et al., 2014).

Straw incorporation in croplands is an effective approach to straw utilization, which provides various kinds of carbon for the microorganisms, and bacterial abundance or structure change would induce the ARGs shifting. In this study, compared to NPK, straw incorporation increased soil organic carbon content. Although straw incorporation did not significantly increase the abundance of bacteria and total ARGs, it changed the abundance of different types of ARGs (Supplementary Table 2). These results indicated that straw incorporation might provide carbon for the growth of soil bacteria, especially that carried ARGs (*qacA_B* and *vanYD*) only detected in RS. Straw incorporation was reported to promote the simultaneous elimination of antibiotics and related ARGs in the paddy soil by changing the dissolvable organic carbon and bacterial structure (Zhang et al., 2022). This study’s results differed from the observed results on microcosmic scales. Therefore, the influence on ARGs should be further explored when straw is returned to the paddy field.

Animal manure is applied into croplands as organic fertilizer to improve soil quality, and the manure carrying antibiotic-resistant bacteria (ARB) and ARGs could simultaneously be transported into agricultural soils, which could increase the abundance and diversity of ARGs in soils (Tiedje et al., 2019; He et al., 2020). In the present observation, compared to NPK, SM significantly increased the diversity, slightly increased the abundance of ARGs in the soil, and gained the highest ARGs detected number across all treatments. These results were similar to the previous studies in which manure amendment dramatically shifted the ARGs patterns in agricultural soils (Zhu et al., 2013; Chen et al., 2017, 2019). The increasing diversity of ARGs in swine-manured soil may be due to several reasons. First, SM is an important host habitat for ARGs carried microbes, which can be directly imported into the soil by fertilization (Zhu et al., 2013). Although not all manure-derived ARGs can persist for a long duration in soil, some of them can still be observed. In this study, eight unique ARGs were only detected in manured soil samples, indicating that manure-borne ARGs were introduced into the agroecosystems (Xie W.Y. et al., 2018). Second, antibiotic resistance bacteria in manure cannot survive in the soil for the long term due to niche shifting, but the native soil bacteria can acquire ARGs through horizontal gene transfer by MGEs, such as transposons, integrons, and broad-host-range plasmids (Jain et al., 1999; Domingues and Nielsen, 2019), which plays an important role in the dissemination of ARGs in various environments. The *int1* gene had the highest relative abundance in the SM-amended soils in this study. These results mean that manure not only introduced novel ARGs to soils but also might introduce or boost MGEs in soil, resulting in the spread of ARGs.

Biochar derived from the pyrolysis of carbon-rich biomass has been widely amended into the soil to increase soil fertility, enhance carbon sequestration, and increase soil water-holding capacity (Xu et al., 2016; Ding et al., 2019; Chen P. et al., 2021). Thus, the changes in soil properties due to biochar amendment might induce the shift of ARGs composition. Due to its high porosity and large surface area, biochar is also applied as an effective adsorbent for the control of soil organic pollutants, such as pesticides and phenols. Biochar can reduce the oxytetracycline and sulfonamide concentrations as well as the corresponding resistance genes (Ye et al., 2016; Duan et al., 2017). Soil biochar amendment was reported to enhance the retention of the ARGs in a former study (He et al., 2021). Furthermore, biochar application has been reported to significantly increase the abundance of bacteria in plastic shed soil (Wang F. et al., 2021), which might change the abundance of ARGs. In this study, compared to NPK, biochar application increased the number of detected ARGs but did not significantly change the relative abundance of ARGs. It might be due to that the special structure of straw-derived biochar provided a special niche and enhanced the soil nutrient (carbon, nitrogen, etc.) contents for the growth of ARGs carried bacteria, which has grown to exceed the detection limits. The improvement of soil organic carbon with biochar amendment was observed from the same field in previous studies (Wang C. et al., 2018; Liu et al., 2021; Wang S. et al., 2021), indicating the biochar might shift the ARGs through increased TOC in paddy soil. Moreover, higher diversity and abundant MGEs were detected in biochar-applied paddy soil than in other treatments; these MGEs contained integrons (*intI1*), insertion sequences (*IS1111*), plasmids (*IncP-oriT*), etc., which mediated the horizontal transfer of ARGs as demonstrated widely in previous research (Post and Hall, 2009; Di Cesare et al., 2016; Kishida et al., 2016). This result indicated a higher frequency of horizontal gene transfer in biochar-applied paddy soil. Therefore, more research needs to be conducted to assess the effects of biochar on the fate of ARGs at the field scale.

### 4.2. Co-occurrence among ARGs, MGEs, and bacterial taxa

Network analysis indicated the co-occurrence patterns among ARGs, MGEs, and bacteria. In this study, the “hub” genes *ermS*, *tetA(P)*, and *mdtG* might be used as the marker genes in the fertilization soils. Furthermore, Module III was composed of several ARGs and MGEs, indicating that these antibiotic-resistance genes might make horizontal gene transfer *via* the assistance of linked MGEs (Zhu et al., 2013).

Soil is an important reservoir of microbes and ARGs. Soil bacteria are the major producers of bioactive substances, such as antibiotics, and at the same time, they are the hosts of numerous ARGs. The network for the relationship between ARGs and bacterial taxa could provide the potential host information of co-occurring ARGs. For instance, *ttgB* and *qacH_351* genes were multidrug resistance genes that were detected in many opportunistic human pathogens (*Pseudomonas aeruginosa*, *Escherichia coli*, and *Acinetobacter baumannii*), and most of these pathogens belong to *Proteobacteria*, but the network showed that *ttgB* and *qacH_351* had many other potential hosts not belong to *Proteobacteria* (The National Center for Biotechnology Information, 2019). Pathogens can acquire various ARGs from pathogens and non-pathogens through horizontal gene transfer *via* MGEs (Forsberg et al., 2012; Wright, 2019), such as integrons, transposons, plasmids, insertion sequences, and phages. The pathogens carrying various ARGs might be developing into “superbugs,” which would further pose a risk to human health *via* direct infection and the food chain (He et al., 2020). Although *Proteobacteria* is one of the dominant phyla in our study, it is weakly correlated with ARGs, indicating that the main host of ARGs in this study may be non-dominant bacteria, and this result was also reported in a previous study (Hu et al., 2017).

### 4.3. Correlations among soil properties, bacteria, MGEs, and ARGs

Soil chemical properties and bacterial characteristics play a pivotal role in shaping ARGs patterns in different croplands (Tiedje et al., 2019; He et al., 2020). In this study, compared with NPK, organic materials input significantly changed the soil properties, especially the content of carbon and nitrogen (Supplementary Table 1), and shifted the profile of ARGs in paddy soils, but showed minor effects on bacterial communities, indicating the resilience of soil indigenous bacteria to the application of the organic material (Macedo et al., 2021). On the one hand, organic materials supply sufficient nutrients for bacterial growth and reproduction, then shift the bacterial abundance and diversity, and finally impact the composition of ARGs. On the other hand, organic matters such as SM might put select pressure on the native soil bacteria due to the heavy metals and/or antibiotics existing, which would then induce the change of ARGs profile. In this study, nitrogen and organic materials input significantly increased the bacterial abundance and abundance of ARGs, and a positive correlation was found between them. This indicated the potential contribution to the increased abundance of some ARGs up to and above the detection limits. This result was inconsistent with a previous study, in which bacterial community structure played a crucial role in shaping the ARGs profile of paddy soil (Xiao et al., 2016). That means the diversity and abundance of ARGs in paddy soils were determined by distinct factors, which need to be further explored.

Organic materials application increased the nutrient content in the soil, which made a contribution to bacterial shifting and then influence the ARGs. Mental test and RDA confirmed that bacterial abundance, TN, and TOC were the major drivers in shaping the profiles of the antibiotic resistome in presently observed soils. That result was consistent with a previous study in farmlands (Cheng et al., 2016), in which positive correlations were observed between the total abundance of ARGs and TN, TP, and TOC. In this study, TN and TOC were significantly correlated with bacterial abundance (Figure 6A), indicating the increment of bacterial abundance with the application of organic materials due to the TN and TOC increment.

In addition, the indigenous bacteria in the soil competed with the exogenous, long-term application of fertilizers, especially livestock manure, stimulated the horizontal gene transfer, and induced the retention and dissemination of ARGs (Udikovic-Kolic et al., 2014). In most previous studies, the critical role of MGEs in the distribution of ARGs had been proved widely. In this study, abundant and diverse MGEs had been detected, and a significant correlation with ARGs was demonstrated by correlation analysis, a co-occurrence network, and the mantel test. This indicated the important potential role of MGEs in shaping the ARGs profile, which may occur *via* horizontal gene transfer (conjugation, transduction, and transformation) as found in a previous study (Hu et al., 2017).

Our study showed that not all organic materials combined with chemical fertilizer increased the diversity and abundance of ARGs significantly, and straw incorporation even reduced the number of resistance genes detected. SM application introduced many exogenous ARGs into paddy soils. In addition, organic materials application, especially biochar addition changed the abundance of the MGEs, which had a positive correlation with ARGs abundance. This indicated the potential horizontal gene transfer playing a crucial role in the spread of ARGs in paddy soils. Thus, it is necessary to consider the risk of ARGs when amending organic materials in paddy fields, for balancing the risks (dissemination of ARGs) and benefits (improvement of soil fertility).

## Data availability statement

The datasets presented in this study can be found in online repositories. The names of the repository/repositories and accession number(s) can be found in the article/Supplementary material.

## Author contributions

ZL: investigation, methodology, and writing—original draft. JPS and FW: writing—original draft. MW: investigation and methodology. JLS: conceptualization, funding acquisition, and writing—review and editing. YL, QZ, and JW: methodology and writing—review and editing. All authors gave the final approval and agreed to be accountable for all aspects of the work.

## Footnotes

1
https://www.biosino.org/node/project/detail/OEP002404
