# Supplementary material for: Impacts of organic materials amendment on the soil antibiotic resistome in subtropical paddy fields
Source: Front Microbiol. 2023 Jan 24;13:1075234. doi: 10.3389/fmicb.2022.1075234 (PMC9904388; doi:10.3389/fmicb.2022.1075234)
Supplement: Supplementary file 1 [file Data_Sheet_1.docx]

Table S1. Soil chemical properties of different treatments

|  | TN  (g/kg) | TP  (g/kg） | TOC  (g/kg) | AP  (mg/kg) | NO_3_^-^-N  (mg/kg) | NH_4_^+^-N  (mg/kg) | C/N | pH |
| --- | --- | --- | --- | --- | --- | --- | --- | --- |
| CK | 1.75±0.08c | 0.62±0.03b | 17.6±0.27c | 12.1±1.36b | 2.15±0.02a | 26.2±1.07b | 10.1±0.42ab | 5.16±0.09b |
| NPK | 1.89±0.05bc | 0.56±0.01b | 18.5±0.33c | 7.79±0.52c | 2.07±0.06ab | 31.9±3.03ab | 9.82±0.09b | 5.20±0.02ab |
| RS | 2.21±0.16a | 0.58±0.07b | 20.6±2.36b | 8.60±0.22c | 1.99±0.04ab | 36.3±3.38a | 9.42±0.64b | 5.33±0.05a |
| SM | 2.17±0.04a | 0.90±0.06a | 20.5±0.56b | 38.0±4.74a | 1.89±0.08b | 31.5±0.38ab | 9.46±0.09b | 5.19±0.05ab |
| BC | 2.09±0.04ab | 0.62±0.03b | 23.4±0.42a | 9.66±0.7bc | 2.12±0.11a | 32.7±3.38ab | 11.2±0.19a | 5.16±0.01b |

TN, total nitrogen; TOC, total organic carbon; AP, available phosphorus; NO_3_^-^-N, nitrate nitrogen; NH_4_^+^-N, ammonium nitrogen. Number composited with the value of mean and standard error, different letters behind the number indicate a significant difference (*P* < 0.05) among different treatments.

Table S2. Mean of detected ARGs relative abundance

|  | CK | NPK | RS | SM | BC |
| --- | --- | --- | --- | --- | --- |
| Aminoglycoside | 9.00E-3±4.17E-4c | 1.09E-2±3.92E-4bc | 1.00E-2±8.55E-4c | 1.24E-2±6.49E-4ab | 1.32E-2±8.42E-4a |
| Amphenicol | 1.84E-4±1.93E-5a | 1.77E-4±8.69E-6a | 1.81E-4±1.24E-5a | 2.08E-4±1.95E-5a | 1.69E-4±1.19E-5a |
| Beta Lactam | 3.54E-3±1.31E-4a | 3.31E-3±7.53E-5a | 2.72E-3±2.70E-4b | 1.62E-3±1.01E-4c | 2.66E-3±1.34E-4b |
| Fluoroquinolone | 3.21E-3±4.25E-4cd | 5.00E-3±2.54E-4ab | 4.38E-3±7.11E-4bc | 2.75E-3±1.61E-4d | 5.86E-3±3.89E-4a |
| MLSB | 7.00E-3±4.19E-4a | 5.62E-3±9.22E-5bc | 5.22E-3±3.83E-4cd | 4.73E-3±2.27E-4d | 6.29E-3±1.36E-4ab |
| Multidrug | 5.72E-3±8.74E-4d | 1.53E-2±1.22E-3b | 1.83E-2±8.32E-4a | 2.03E-2±1.04E-3a | 1.19E-2±4.42E-4c |
| Phenicol | 1.83E-4±8.38E-6a | 1.12E-4±1.88E-5c | 1.48E-4±1.26E-5b | 1.07E-4±8.05E-6c | 1.01E-4±6.05E-6c |
| Sulfonamide | -- | 6.47E-4±1.13E-4a | 1.47E-4±5.39E-5d | 5.99E-4±1.80E-4b | 1.54E-4±3.52E-5c |
| Tetracycline | 2.39E-3±1.02E-4d | 3.36E-3±1.20E-4c | 3.83E-3±1.11E-4b | 4.42E-3±1.69E-4a | 2.10E-3±6.45E-5d |
| Trimethoprim | 1.40E-4±1.64E-5a | 1.29E-4±1.66E-5a | 1.11E-4±1.68E-5a | 1.26E-4±1.26E-5a | 1.27E-4±1.12E-5a |
| Vancomycin | 4.42E-4±5.12E-5a | 4.05E-4±3.32E-5a | 3.42E-4±5.89E-5a | 3.34E-4±2.82E-5a | 3.83E-4±1.82E-5a |
| Others | 1.34E-3±1.07E-4a | 8.86E-4±8.55E-5b | 6.15E-4±8.37E-5c | 4.33E-4±3.08E-5c | 4.79E-4±4.09E-5c |

Number composited with the value of mean and standard error, different letters behind the number indicate a significant difference (*P* < 0.05) among different treatments.


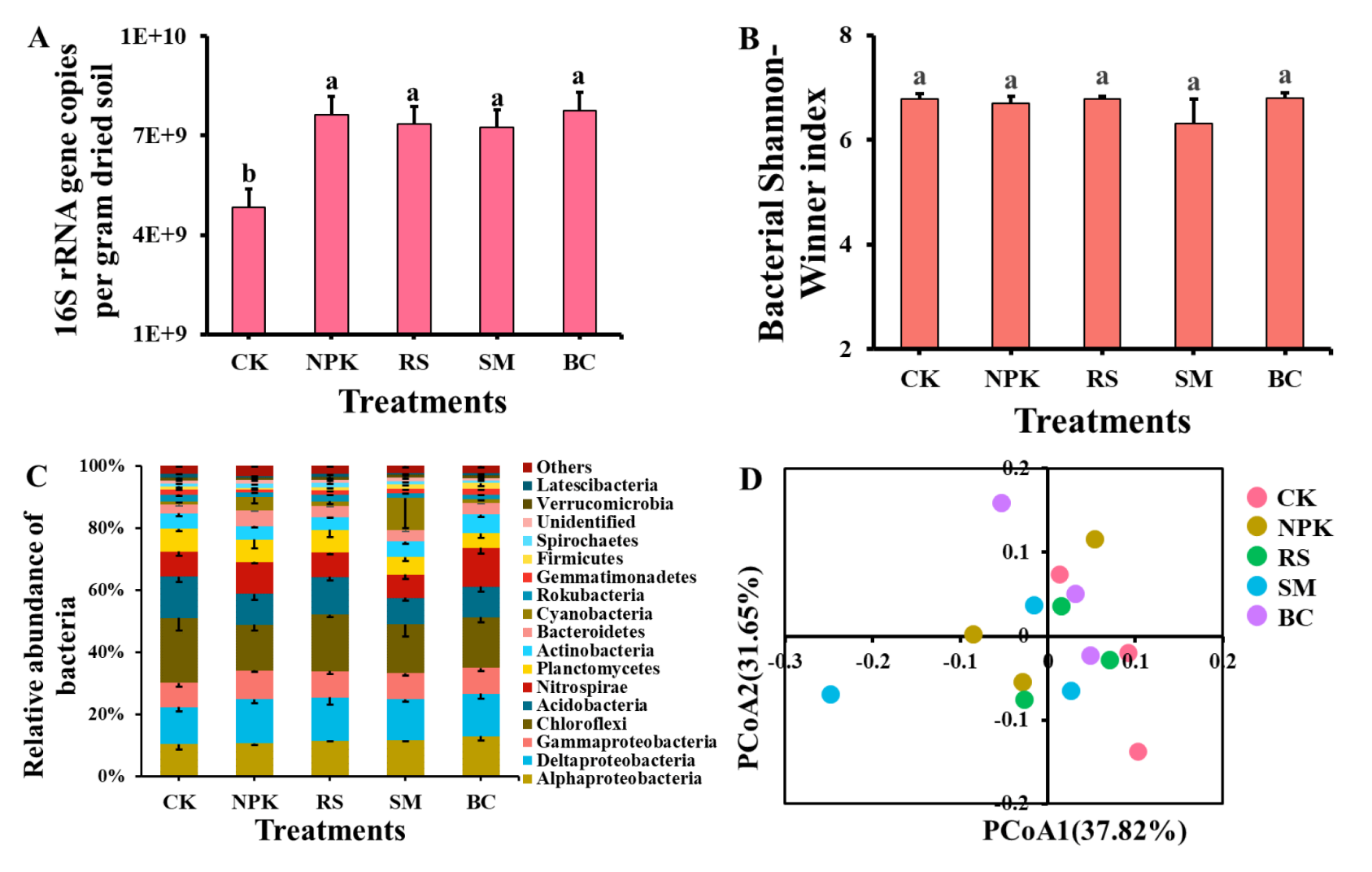


Fig S1. Bacterial 16S rRNA gene copies (A), Shannon-Winner index (B), community structure (C) and the PCoA analysis based on the relative abundance of bacterial community using Bray–Curtis distances (D).
